# Zebrafish Larvae Are a Suitable Model to Investigate the Metabolic Phenotype of Drug-Induced Renal Tubular Injury

**DOI:** 10.3389/fphar.2018.01193

**Published:** 2018-10-16

**Authors:** Judit Morello, Rico J. E. Derks, Susana S. Lopes, Evelyne Steenvoorden, Emilia C. Monteiro, Oleg A. Mayboroda, Sofia A. Pereira

**Affiliations:** ^1^CEDOC, Chronic Diseases Research Centre, NOVA Medical School/Faculdade de Ciências Médicas, Universidade NOVA de Lisboa, Lisbon, Portugal; ^2^Center for Proteomics and Metabolomics, Leiden University Medical Centre, Leiden, Netherlands; ^3^Department of Chemistry, Tomsk State University, Tomsk, Russia

**Keywords:** translational models, zebrafish, metabolomics, renal tubular toxicity, mitochondria, kidney injury biomarkers

## Abstract

Prevention and treatment of drug-induced renal injury (DIRI) rely on the availability of sensitive and specific biomarkers of early kidney injury and predictive animal models of human pathophysiology. This study aimed to evaluate the potential of zebrafish larvae as translational model in metabolic profiling of DIRI. Zebrafish larvae were exposed to the lethal concentration for 10% of the larvae (LC10) or ½ LC10 of gentamicin, paracetamol and tenofovir as tenofovir disoproxil fumarate (TDF) and tenofovir (TFV). Metabolites were extracted from whole larvae and analyzed by liquid chromatography-mass spectrometry. Principal component analysis showed that drug exposition to the LC10 of paracetamol, TFV, and TDF was the main source of the variance of the data. To identify the metabolites responsible for the toxic effects of the drugs, partial least squares discriminant analyses were built between the LC10 and ½ LC10 for each drug. Features with variable importance in projection> 1.0 were selected and Venn diagrams were built to differentiate between the common and drug specific metabolites of DIRI. Creatine, tyrosine, glutamine, guanosine, hypoxanthine were identified as common metabolites, adenosine and tryptophan as paracetamol-specific and xanthine and oxidized glutathione as tenofovir-specific. Those metabolic changes can be associated with alterations in energy metabolism, xenobiotic detoxification and protein catabolism, all described in the human pathophysiology of DIRI. Thus, zebrafish proved to be a suitable model to characterize the metabolic changes associated with DIRI. This information can be useful to early diagnose DIRI and to improve our knowledge on the mechanisms of DIRI.

## Introduction

Drug toxicity is the principal cause of attrition during drug development (Waring et al., 2015) and is one of the leading causes of morbidity and mortality (Pirmohamed et al., 2004). In particular, DIRI is responsible for 19–25% of all cases of acute kidney injury (Mehta et al., 2004). The kidney is one of the principal targets of drug toxicity but the current tests to measure renal function such as serum creatinine and blood urea nitrogen are not sensitive enough to detect early kidney injury (Slocum et al., 2012). Therefore, novel screening tools complementary to traditional biomarkers are of utmost importance. Furthermore, a better understanding of the pathophysiology of DIRI is critical for uncovering new therapeutic strategies that may prevent or modify the disease progression.

There are two main reasons for the increasing interest of toxicologists in metabolomics. Firstly, metabolomics can be used to find signatures of toxicity that can be useful as diagnostic biomarkers and/or to predict toxicity during drug development. Secondly, metabolomics can improve our understanding on the mechanisms of drug toxicity through the identification of the cellular pathways that are affected by the toxicants (Bouhifd et al., 2013). The *COMET* and *MetaMap*^®^*To*x projects are two examples of the potential of toxicometabolomics as a drug toxicity screening tool. Those studies generated metabolic databases from urine and plasma samples of rats exposed to different toxicants that were further used to predict specific organ toxicity, including DIRI (Ebbels et al., 2007; van Ravenzwaay et al., 2012; Mattes et al., 2013). Toxicometabolomics proved also to be useful to identify new biomarkers and/or elucidate the mechanisms underlying DIRI. Most of the studies evaluated cisplatin or gentamicin-associated tubular toxicities. The metabolites that were commonly affected were characteristic of Fanconi’s syndrome (amino acids, glucose, phosphates), tricarboxylic acid cycle metabolites and tryptophan metabolites (Lenz et al., 2005; Xu et al., 2008; Boudonck et al., 2009; Zhang et al., 2016). Interestingly, some of the identified metabolites preceded the changes in serum creatinine levels, which illustrates the predictive power of metabolomics in detecting early renal insult (Portilla et al., 2006).

The bulk of the toxicity data was obtained using murine models. Zebrafish larvae are a strong alternative animal model because of their numerous advantageous features such as rapid development, small size, easy and fast chemical administration, transgenic capabilities, low financial cost and high-throughput capabilities (McGrath and Li, 2008; Peterson and Macrae, 2012). The feasibility of zebrafish larvae for the evaluation of DIRI has recently been demonstrated. A detailed analysis on the functional and morphological renal alterations induced by gentamicin, paracetamol and tenofovir in zebrafish larvae showed tubular alterations similar to those described in man. In addition, electron microscopy revealed that tubular mitochondria were affected by the tested drugs, which points to severe metabolic alterations (Gorgulho et al., 2018). This evidence led us to hypothesize that zebrafish larvae are an attractive model for toxicometabolomics studies.

To test this hypothesis, an untargeted metabolomics study was herein performed in zebrafish larvae exposed to drugs with different mechanisms of nephrotoxicity. The present study gives for the first time a comprehensive evaluation of the metabolic alterations associated with drug-induced renal tubular toxicity in zebrafish larvae.

## Materials and Methods

### Ethics Statement

All procedures involving zebrafish were approved by the Ethical Committee of the Instituto Gulbenkian de Ciência (IGC) number A001.2014, according with the directives from Direcção Geral Veterinária (Portaria n° 1005/92 de 23 de Outubro).

### Zebrafish Larvae

We used the zebrafish line Tg(wt1b:GFP, cdh17:GFP); mitfa^-/-^; roy^-/+^^or^^+/+^ because it was the one that was previously used to define the lethality curves and characterize the renal tubular effects of gentamicin, paracetamol, and tenofovir (Gorgulho et al., 2018). Drug susceptibility might change among different zebrafish lines. Thus, using a different zebrafish line (i.e., wild-type zebrafish) would have implied the use of more animals to define the new lethality curves and to characterize the renal damage. In order to reduce the number of animals (one of the three Rs of the Good Animal Practices in Science), we decided to use the zebrafish line that was previously associated with a phenotype of DIRI at the concentrations used in this study.

Three aquariums of 10 male and 10 female adult zebrafish were grown at the CEDOC Fish Facility of the NOVA Medical School, Lisbon, Portugal. Male and female fish from each aquarium were mated once per week according to the protocols of the Fish Facility. Embryos were grown at 28°C in standard E3 solution plus 10 mM HEPES buffer till 4 days post fertilization (dpf), time point at which zebrafish pronephros is completely mature (Kramer-Zucker et al., 2005).

### Drugs

Three drugs known to cause tubular damage in man and proved to cause renal damage in zebrafish larvae by us and others were tested (Hentschel et al., 2005; Peng et al., 2010; Rider et al., 2012; Cianciolo Cosentino et al., 2013; Westhoff et al., 2013; Gorgulho et al., 2018): gentamicin sulfate, paracetamol (Sigma-Aldrich) and tenofovir (Sequoia Research Products, United Kingdom). Tenofovir was administered as the prodrug tenofovir disoproxil fumarate (TDF) and as tenofovir (TFV), the form of the drug that is present in the blood after TDF hydrolysis by esterases.

Stock solutions were prepared for all drugs at solubility concentrations: gentamicin 5,000 μg⋅mL^-1^, paracetamol 12,000 μg⋅mL^-1^, TDF 6,000 μg⋅mL^-1^ and TFV 5,000 μg⋅mL^-1^. All drugs except TFV were dissolved in sterile distilled water. TFV was prepared in embryo media. All stock solutions were aliquoted and stored at -20°C until further use.

### Drug Administration and Sampling

Fifty zebrafish larvae of 4 dpf were transferred to each well of six-well plates. Embryo media was completely removed from each well and immediately after that, specific volumes of embryo media and drug stock solution or drug vehicle (negative controls) were added to each well to achieve the desired drug concentration (total volume per well = 8.75 mL).

Drugs were delivered into the embryo media at two different concentrations: lethal concentration for 10% of the zebrafish larvae (LC10) associated with functional and/or morphological damage in our previous study (Gorgulho et al., 2018), and a non-lethal concentration equal to ½ of LC10. To confirm the LC10 concentrations, three concentrations close to the original LC10 values were tested for each drug in 10 zebrafish larvae in triplicate. The new LC10 were not different from the ones used in our previous article (Gorgulho et al., 2018). In ascending order, the LC10 were: 521 μM for gentamicin, 2,676 μM for TDF, 9,400 μM for TFV and 19,185 μM for paracetamol.

All experiments were performed in 5–10 replicates during 17 consecutive days. One negative control was always run in parallel in the same plate to check the viability of the zebrafish larvae. The volume of drug vehicle that was added to each negative control was equivalent to the volume of the corresponding drug stock solution. In this way, negative controls were named as controls LC10 when they received the same volume of drug vehicle as LC10 positive samples and controls ½ LC10 when they received the same volume of drug vehicle as ½ LC10 positive samples. The lethality of negative controls was always 0%.

Upon 24 h of drug exposure, dead larvae were discarded and 40 larvae were transferred from each well to a clean microcentrifuge tube. Larvae were immediately washed four times with cold water to remove drugs and/or drug metabolites. After that, larvae were euthanized by rapid chilling to remove all remaining embryo media before snap freezing in liquid nitrogen to quench any enzymatic activity. A total of 83 samples were stored at -20°C till extraction (*n* = 10 gentamicin LC10, *n* = 10 gentamicin ½ LC10, *n* = 10 paracetamol LC10, *n* = 10 paracetamol ½ LC10, *n* = 10 TDF LC10, *n* = 10 TDF ½ LC10, *n* = 5 TFV LC10, *n* = 5 TFV ½ LC10, *n* = 6 negative controls LC10, *n* = 7 negative controls ½ LC10).

### Metabolite Extraction and LC-MS Acquisition

Samples were randomized before extraction. All samples were extracted on the same day in four blocks of 17–24 samples each one. Metabolite extraction from whole zebrafish larvae was performed with methanol:water (Huang et al., 2013). Briefly, 350 μL of cold methanol:water 2:1 were added to each microcentrifuge tube containing 40 larvae of zebrafish. Each sample was vortexed for 3 min and placed in the ultrasound for 15 min at 4°C to enhance metabolite extraction. Then, samples were centrifuged for 10 min at maximum speed and 140 μL of the supernatant was transferred to a new microcentrifuge tube for dry-vacuum. Dried samples were reconstituted with 70 μL of water. 10 μL of each reconstituted sample were pooled (quality control pool; QC pool) and injected throughout sample acquisition to check the performance of the LC-MS. 55 μL of each sample or QC pool were injected into the RPLC-Q-TOF (Ultimate 3000 RS tandem Ultra High Performance Chromatography system, Dionex, Amsterdam, Netherlands; Electrospray Ionization – Ultra High Resolution -Time of Flight maXis, Bruker Daltonics, Bremen, Germany). The details of the RPLC-Q-TOF method have previously been reported (Nevedomskaya et al., 2011; Pacchiarotta et al., 2012).

### Pre-processing of LC-MS Data

Pre-processing of raw data from 83 samples and 12 QC pools consisted of alignment of retention time, peak picking, peak matching, filtering and normalization.

Retention time was aligned using the msalign package with a mass error of 5 ppm (Nevedomskaya et al., 2009). Peak picking and peak matching were done using the XCMS package (Smith et al., 2006). The values of the parameters for peak picking were: mass error = 5 ppm, S/N = 10, Noise = 15,000, peak width = 5–20s, prefilter = 2, 15,000 and scan range = 30–420s. The values of the parameters for peak matching were bandwidth = 2, presence of the peak in all QC pools and m/z width = 0.1. A final filter was applied to keep only those features with a relative standard deviation≤ 0.3 among the QC pool. The final list contained 445 features. Data was normalized by total area followed by probabilistic quotient normalization (Dieterle et al., 2006). Data was centered and unit variance scaled before statistical analysis.

### Exclusion of Exogenous Compounds

The signals from exogenous compounds (HEPES and drug related compounds) were clearly seen in the chromatograms. Those signals were excluded from the list of 445 features with the following strategy: (1) exclusion of those ions matching the theoretical *m/z* and isotope patterns of HEPES and drug related compounds (more details in our previous article) (Gorgulho et al., 2018); (2) exclusion of those features highly correlated (coefficient of correlation ≥ 0.9) with the features excluded in the 1st step. The 2nd step assures the complete exclusion of adducts (i.e., with Na+ or K+), fragments and isotopes from all exogenous compounds from the list of 445 features.

### Statistical Analysis

Multivariate analysis was performed using principal component analysis (PCA) with R (Team RDC, R Foundation for Statistical Computing). Classifiers of drug toxicity were selected using partial least square – discriminant analysis (PLS-DA) with SIMCA 12 + software package (Umetrics, Umeå, Sweden).

### Metabolite Identification

Metabolites annotation was carried out according to the guidelines for the minimal reporting standards (Sumner et al., 2007). The Smart Formula tool within the Data Analysis software (version 4.1) was used for the initial ion annotation based on accurate mass (mass error< 5 ppm) and isotopic distribution (sigma value< 20). The results were matched against online metabolomics databases (METLIN, Human Metabolome Database, MassBank). Hits were confirmed with MS–MS experiments of the sample with the highest intensity for each of the putative metabolites and the reference standards. MS–MS experiments were performed on the same RPLC-Q-TOF instrument in auto MS–MS mode.

## Results

### Quality of the Data

**FIGURE 1:**
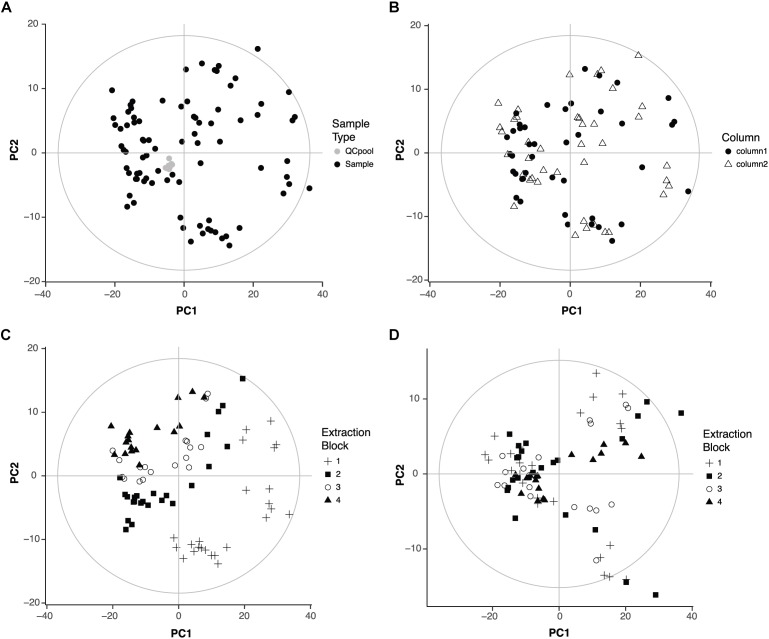
Quality of the data. **(A)** Score plot of the PCA of the 95 samples that were analyzed (zebrafish samples, *n* = 83, QC pool, *n* = 12). First two components covered 47 and 12% of the variation of the data, respectively. **(B)** Score plot of the PCA of the 83 zebrafish samples showing the influence of the chromatographic column. First two components covered 48 and 12% of the variation of the data, respectively. **(C)** Score plot of the PCA of the 83 zebrafish samples showing the influence of the extraction block. **(D)** Score plot of the PCA after correction for the influence of the extraction block. First two components cover 46 and 8% of the variation of the data, respectively.

After LC-MS data pre-processing, the first step in any metabolomics experiment is to analyze the quality of the data. This step checks if analytical factors related for example with sample collection, metabolite extraction or sample acquisition have any influence in the metabolic profile. PCA is a very useful method to address this question because it allows to identify the variables that are responsible for the main differences among samples in an unbiased fashion. Thus, a PCA model was built with all the samples and the QC pools that were repeatedly injected throughout the data acquisition (**Figure 1A**). The clustering of the QC pools indicates there are no metabolic differences among QC pools and thus, proves the good performance of the LC-MS. The first two components of the PCA covered 59% of the variance in the data.

Then, a PCA model was built without the QC pools to check the influence of two other analytical factors: the chromatographic column and the metabolite extraction. Our LC method uses two analytical columns that work alternatively to speed up the acquisition (Nevedomskaya et al., 2011; Pacchiarotta et al., 2012). Thus, potential differences between columns might have an influence in the data. Regarding the metabolite extraction, since samples were organized in four groups or blocks of extraction, it was important to check if there were metabolic differences among those blocks. The first two components of the new PCA model explained 60% of the variance in the data. The score plot was colored according to the chromatographic column or extraction block. The column did not influence the distribution of the samples (**Figure 1B**). However, the extraction block had an important influence: samples extracted in the first block appeared far away from the samples extracted in the fourth block (**Figure 1C**).

The influence of the extraction block might interfere with the effects of the drugs on the metabolic profile, the aim of this study. Thus, the following strategy was adopted to correct the effect of the extraction block considering that not all variables were equally affected and all different conditions (drug and concentration) were present in all extraction blocks. For each variable, both the mean per extraction block (block mean) and the total mean (overall mean) were calculated. A correction factor was then calculated as the quotient block mean/overall mean. Following this strategy, the correction factor was different for each variable (and for each extraction block). After applying the correction factor, the influence of the extraction was no longer visible in the PCA (**Figure 1D**). The first two components explained 54% of the variance of the data.

### Influence of the Drug and Concentration on the Metabolic Profile

**FIGURE 2:**
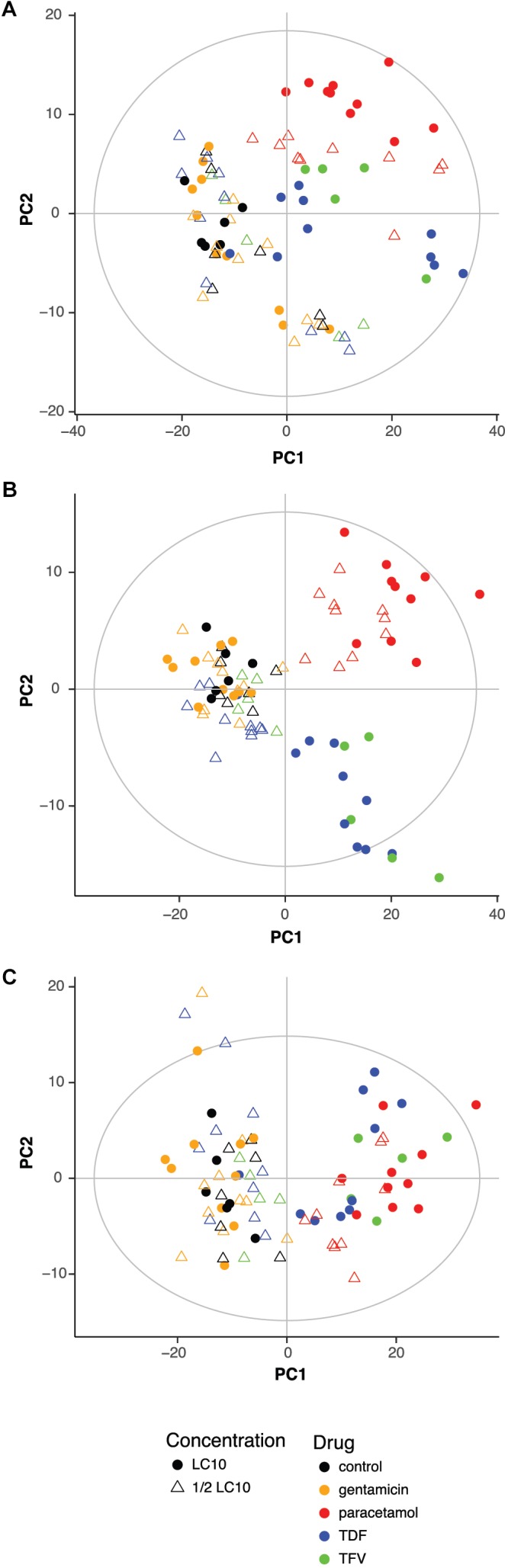
Influence of drug and concentration on the metabolic profile. Score plot of the PCA of the 83 zebrafish samples without **(A)** and with correction **(B)** of extraction block effect showing the influence of drug and concentration. **(C)** Score plot of the PCA of the 83 zebrafish samples after correction for the extraction block effect and after exclusion of exogenous compounds effect showing the influence of drug and concentration. LC10, lethal concentration for 10% of zebrafish larvae; TDF, tenofovir disoproxil fumarate; TFV, tenofovir.

To evaluate if drugs had any influence on the metabolic profile, previous PCA scores plots representing the 83 samples were characterized according to the drug and the concentration. PCA showed a clear effect of the drugs with or without correcting for the influence of the extraction (**Figures 2A,B**). Zebrafish larvae exposed to paracetamol LC10 and ½ LC10, TDF LC10 and TFV LC10 were far from the negative controls, while gentamicin LC10 and ½ LC10, TDF ½ LC10 and TFV ½ LC10 appeared close to the negative controls. This distribution reflects a concentration dependent effect in the PCA: the lowest concentrations, corresponding to both ½ LC10 and LC10 of gentamicin and ½ LC10 of TDF, were the closest ones to the negative controls. Only concentrations higher than 2,676 μM, which corresponds to TDF LC10, showed a different metabolic profile when compared to the negative controls. The only exception to this rule was the TFV ½ LC10 of 4,700 μM, which appeared close to the negative controls.

Other interesting effect seen in the score plot of the PCA is that TDF LC10 and TFV LC10 appeared together separated from paracetamol, which can reflect the differences in drug related compounds between the two clusters.

The next step was to exclude all exogenous compounds from the 445 features. A total of 22 features were excluded and their m/z corresponded to HEPES and drug related compounds (**Supplementary Table S1**). **Figure 2C** shows the score plot of a PCA model built after exclusion of these variables. As in **Figure 2B**, the only samples that showed a different metabolic profile from the negative controls, were the ones exposed to LC10 TDF, LC10 TFV, ½ LC10 paracetamol and LC10 paracetamol. One very interesting change was that the differences between paracetamol and TDF and TFV were no longer visible. Moreover, the cluster of samples that were grouped with the negative controls showed more heterogeneity.

### Metabolic Markers of Drug Toxicity

Principal component analysis showed a clear influence of the toxic concentrations of paracetamol, TDF, and TFV on the metabolic profile (**Figures 2A–C**). In order to identify the metabolites related to the toxic effects of the drugs, PLS-DA was applied with the treatment group as response variable (*Y* variable). Paired PLS-DA models were created between negative controls and ½ LC10, negative controls and LC10 and LC10 and ½ LC10 for each drug. This approach was chosen since the interpretation of a two-class PLS-DA model is easier than the one with multiple classes. As already seen in the PCA, gentamicin PLS-DA models failed to show any separation between negative controls, gentamicin LC10 or gentamicin ½ LC10 so we decided to exclude gentamicin samples from the rest of the analysis (**Supplementary Table S2** and **Supplementary Figure S1**).

**FIGURE 3:**
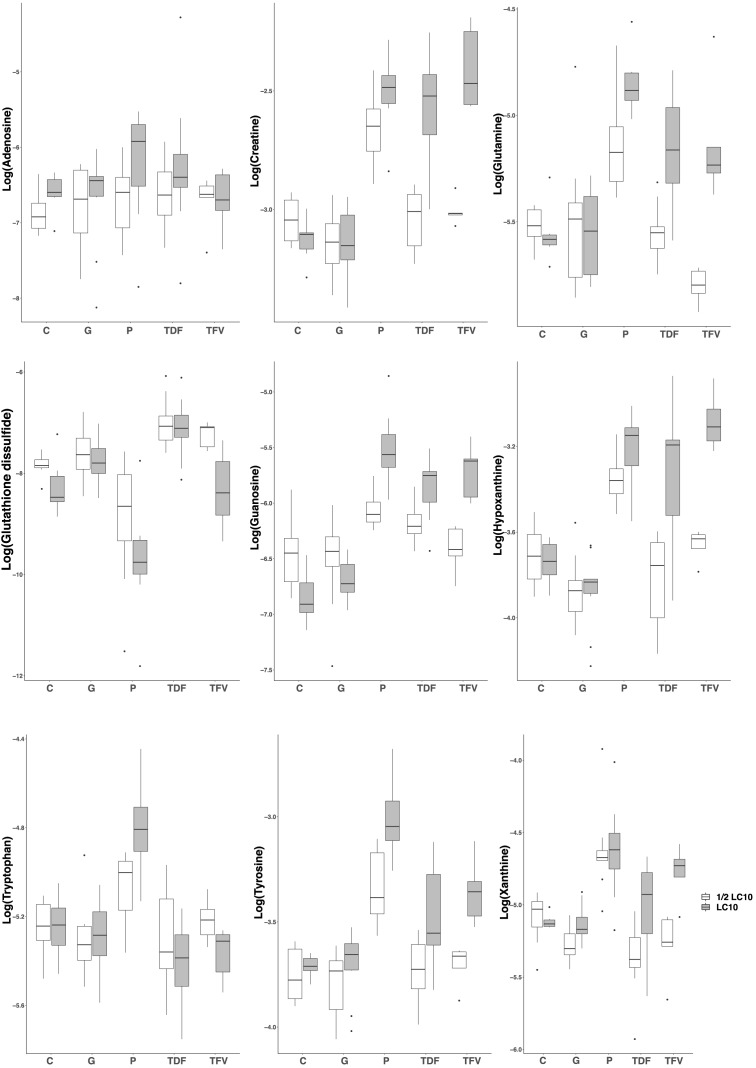
Boxplots of the identified metabolites after PLS-DA modeling. Creatine, glutamine, guanosine, hypoxanthine and tyrosine were associated with the renal tubular toxicity of paracetamol, TDF and TFV. Adenosine and tryptophan were associated with the renal tubular toxicity of paracetamol. Xanthine and glutathione disulfide were associated with the renal tubular toxicity of TFV. LC10, lethal concentration for 10% of zebrafish larvae; C, control, G, gentamicin; P, paracetamol; TDF, tenofovir disoproxil fumarate; TFV, tenofovir.

In order to reveal the metabolites responsible for the toxic effects of the drugs, we chose the PLS-DA models between LC10 and ½ LC10 because they are the only ones that can discriminate between the toxic effects associated with the LC10 in our previous study and the non-lethal effects of ½ LC10 considered as non-toxic in this study. From each of the three PLS-DA models between LC10 and ½ LC10, we selected those features with the variable importance on the projection (VIP) value> 1.0 on the first component, the one that was responsible for the separation of the samples (*n* = 188 for paracetamol, 222 for TDF and 249 for TFV). To differentiate between the common and drug specific metabolites associated with the toxic effects of the drugs, we built Venn diagrams with the selected features. A total of 119 features were common for the three drugs and 23, 25, and 30 were specific for paracetamol, TDF, and TFV, respectively. Compound assignment was successful for nine metabolites: guanosine, hypoxanthine, creatine, glutamine and tyrosine as common metabolites, adenosine and tryptophan as metabolites specific of paracetamol toxicity and xanthine and glutathione disulfide as metabolites specific of TFV toxicity (**Supplementary Table S3** and **Supplementary Figure S2**). All metabolites except glutathione disulfide presented higher concentrations in zebrafish exposed to LC10 than in zebrafish exposed to ½ LC10 (**Figure 3** and **Supplementary Figure S2**).

## Discussion

This current study gives for the first time a detailed evaluation of the metabolic alterations associated with drug-induced renal tubular injury in zebrafish larvae.

For practical reasons, our experiments were performed on whole larvae and not on isolated nephrons. Thus, we cannot exclusively associate the observed metabolic changes with the renal tubule. Even if all tested drugs affected the renal tubule, it is possible that they might have also damaged other organs (i.e., paracetamol is well known to cause liver toxicity). This plausible non-renal toxicity does not lessen the power of our approach. In fact, the kidneys play a key role in the metabolic regulation of an organism; they control the excretion of metabolic wastes, the body pH, the extracellular volume and the production and secretion of hormones. Thus, even at very early stages, kidney injury might induce not only organ-specific but global metabolic changes (Lane et al., 2013). Those global metabolic changes and/or non-renal toxicity-related metabolic changes might also contribute to DIRI. In this way, our approach, that reflects what happens in the whole organism, seems to be more valid in terms of translational model of DIRI.

Our study revealed changes in metabolites of purines, glutathione and amino acids upon exposure to tubular neurotoxicants. Similar alterations were also described in plasma and kidney tissue of rats exposed to different drugs associated with tubular damage (Boudonck et al., 2009; Zgoda-Pols et al., 2011; Mattes et al., 2013). Those metabolic changes can be linked with alterations in energy metabolism, xenobiotic metabolism and protein catabolism, which have already been described in the pathophysiology of acute kidney injury (Druml, 2013).

Proximal tubular cells depend on oxidative phosphorylation to meet the energetic demand of the basolateral Na^+^/K^+^ ATPase in the reabsorption of glucose and ions (Basile et al., 2012). The drugs herein used have been described to induce tubular mitochondrial swelling and/or cristae fragmentation in zebrafish larvae (Gorgulho et al., 2018), signs that can be associated with a functional inhibition of ATP synthesis (Galloway and Yoon, 2012). The consequent increase in consumption of ATP, which occurs in parallel with GTP depletion (Dagher, 2000), might explain the observed increase in the degradation products of purine nucleotides (adenosine, guanosine, hypoxanthine and xanthine). In addition, the depletion of ATP might activate the renal energy deposit of phosphocreatine. Phosphocreatine works as energy source facilitating the recycling of ATP (Wyss and Kaddurah-Daouk, 2000), which can be translated into the observed increase in creatine levels.

On the other hand, the inhibition of oxidative phosphorylation leads also to a burst of reactive oxygen species (Adam-Vizi and Chinopoulos, 2006) and a consumption of antioxidant defenses. A decrease in oxidized glutathione was observed, which might indicate a decrease in glutathione synthesis (Santangelo et al., 2004; Shang et al., 2016) or an increase in conjugation to highly reactive compounds (Wang and Ballatori, 1998).

Finally, one of the leading metabolic alterations of acute kidney injury is the activation of protein catabolism with excessive release of amino acids from skeletal muscle (Flügel-Link et al., 1983). That might explain the increase observed in some amino acids in this study (glutamine, tyrosine and tryptophan).

The design of our study that included exposure to lethal and nonlethal concentrations allowed us to selectively detect those metabolites associated with the toxic effects of all drugs except for gentamicin. Zebrafish exposed to both LC10 and ½ LC10 of gentamicin had the same metabolic profile as untreated larvae. In our previous study, zebrafish exposed to the LC10 of gentamicin experienced renal alterations including a decrease in the clearance of inulin and mitochondrial morphological alterations (Gorgulho et al., 2018). However, we reported difficulties to detect gentamicin in zebrafish larvae and actually the chromatograms of the whole larvae exposed to gentamicin or water looked exactly the same (Gorgulho et al., 2018). In this present work, we do not have other explanation than the instrumental one to explain this result but we consider important to report.

Tenofovir was the drug with lowest potency for metabolic changes. This can be explained by the lower bioavailability of TFV compared with its prodrug TDF (Shaw et al., 1997). The low lipophilicity of TFV implies the administration of higher concentrations of TFV than of TDF to reach similar blood concentrations.

## Conclusion

This work demonstrates the usefulness of zebrafish larvae to characterize the metabolic changes associated with tubular toxic drugs. Purine metabolites, oxidized glutathione and amino acids were identified to be associated with the toxic effects of paracetamol and/or tenofovir. Those metabolites can be associeted with the pathophysiology of renal tubular injury, mainly affecting the mitochondria, and might be useful for an early diagnosis of DIRI, for going deeper in the underlying mechanisms of DIRI and for screening nephroprotective compounds.

## Author Contributions

JM, SL, EM, SP, and OM designed the study. JM and ES performed the experiments. JM, RD, and OM analyzed the data. JM, SL, OM, and SP wrote the paper.

## Conflict of Interest Statement

The authors declare that the research was conducted in the absence of any commercial or financial relationships that could be construed as a potential conflict of interest.

## Abbreviations

DIRI: drug-induced renal injury
LC10: lethal concentration for 10% of the population
PCA: principal component analysis
PLS-DA: partial least square – discriminant analysis
QC: quality control
RPLC-Q-TOF: reverse phase liquid chromatography – quadruple – time of flight
TDF: tenofovir disoproxil fumarate
TFV: tenofovir

## References

[B1] Adam-Vizi V. Chinopoulos C. (2006). Bioenergetics and the formation of mitochondrial reactive oxygen species. *Trends Pharmacol. Sci.* 27 639–645. 10.1016/j.tips.2006.10.005 17056127

[B2] Basile D. P. Anderson M. D. Sutton T. A. (2012). Pathophysiology of acute kidney injury. *Compr. Physiol.* 2 1303–1353. 10.1002/cphy.c110041 23798302 PMC3919808

[B3] Boudonck K. J. Mitchell M. W. Német L. Keresztes L. Nyska A. Shinar D. (2009). Discovery of metabolomics biomarkers for early detection of nephrotoxicity. *Toxicol. Pathol.* 37 280–292. 10.1177/0192623309332992 19380839

[B4] Bouhifd M. Hartung T. Hogberg H. T. Kleensang A. Zhao L. (2013). Review: toxicometabolomics. *J. Appl. Toxicol.* 33 1365–1383. 10.1002/jat.2874 23722930 PMC3808515

[B5] Cianciolo Cosentino C. Skrypnyk N. I. Brilli L. L. Chiba T. Novitskaya T. Woods C. (2013). Histone Deacetylase inhibitor enhances recovery after AKI. *J. Am. Soc. Nephrol.* 24 943–953. 10.1681/ASN.2012111055 23620402 PMC3665399

[B6] Dagher P. C. (2000). Modeling ischemia in vitro: selective depletion of adenine and guanine nucleotide pools. *Am. J. Physiol. Physiol.* 279 C1270–C1277. 10.1152/ajpcell.2000.279.4.C1270 11003607

[B7] Dieterle F. Ross A. Schlotterbeck G. Senn H. (2006). Probabilistic quotient normalization as robust method to account for dilution of complex biological mixtures. Application in 1H NMR metabonomics. *Anal. Chem.* 78 4281–4290. 10.1021/ac051632c 16808434

[B8] Druml W. (2013). *Nutritional Management of Acute Kidney Injury.* Amsterdam: Elsevier Inc.

[B9] Ebbels T. M. D. Keun H. C. Beckonert O. P. Bollard M. E. Lindon J. C. Holmes E. (2007). Prediction and classification of drug toxicity using probabilistic modeling of temporal metabolic data: the consortium on metabonomic toxicology screening approach. *J. Proteome Res.* 6 4407–4422. 17915905 10.1021/pr0703021

[B10] Flügel-Link R. M. Salusky I. B. Jones M. R. Kopple J. D. (1983). Protein and amino acid metabolism in posterior hemicorpus of acutely uremic rats. *Am. J. Physiol.* 244 E615–E623. 10.1152/ajpendo.1983.244.6.E615 6305204

[B11] Galloway C. A. Yoon Y. (2012). What comes first, misshape or dysfunction? The view from metabolic excess. *J. Gen. Physiol.* 139 455–463. 10.1085/jgp.201210771 22641640 PMC3362522

[B12] Gorgulho R. Jacinto R. Lopes S. S. Pereira S. A. Tranfield E. M. Martins G. G. (2018). Usefulness of zebrafish larvae to evaluate drug-induced functional and morphological renal tubular alterations. *Arch. Toxicol.* 92 411–423. 10.1007/s00204-017-2063-1 28932931

[B13] Hentschel D. M. Park K. M. Cilenti L. Zervos A. S. Drummond I. Bonventre J. V. (2005). Acute renal failure in zebrafish: a novel system to study a complex disease. *Am. J. Physiol. Renal Physiol.* 288 F923–F929. 10.1152/ajprenal.00386.2004 15625083

[B14] Huang S. M. Xu F. Lam S. H. Gong Z. Ong C. N. (2013). Metabolomics of developing zebrafish embryos using gas chromatography- and liquid chromatography-mass spectrometry. *Mol. Biosyst.* 9 1372–1380. 10.1039/c3mb25450j 23475132

[B15] Kramer-Zucker A. G. Wiessner S. Jensen A. M. Drummond I. A. (2005). Organization of the pronephric filtration apparatus in zebrafish requires Nephrin, Podocin and the FERM domain protein Mosaic eyes. *Dev. Biol.* 285 316–329. 10.1016/j.ydbio.2005.06.038 16102746 PMC2836015

[B16] Lane K. Dixon J. J. MacPhee I. A. M. Philips B. J. (2013). Renohepatic crosstalk: does acute kidney injury cause liver dysfunction? *Nephrol. Dial. Transplant.* 28 1634–1647. 10.1093/ndt/gft091 23685679

[B17] Lenz E. M. Bright J. Knight R. Westwood F. R. Davies D. Major H. (2005). Metabonomics with 1H-NMR spectroscopy and liquid chromatography-mass spectrometry applied to the investigation of metabolic changes caused by gentamicin-induced nephrotoxicity in the rat. *Biomarkers* 10 173–187. 10.1080/13547500500094034 16076731

[B18] Mattes W. B. Kamp H. G. Fabian E. Herold M. Krennrich G. Looser R. (2013). Prediction of clinically relevant safety signals of nephrotoxicity through plasma metabolite profiling. *Biomed. Res. Int.* 2013:202497. 10.1155/2013/202497 23762827 PMC3673329

[B19] McGrath P. Li C.-Q. (2008). Zebrafish: a predictive model for assessing drug-induced toxicity. *Drug Discov. Today* 13 394–401. 10.1016/j.drudis.2008.03.002 18468556

[B20] Mehta R. L. Pascual M. T. Soroko S. Savage B. R. Himmelfarb J. Ikizler T. A. (2004). Spectrum of acute renal failure in the intensive care unit: the PICARD experience. *Kidney Int.* 66 1613–1621. 10.1111/j.1523-1755.2004.00927.x 15458458

[B21] Nevedomskaya E. Derks R. Deelder A. M. Mayboroda O. A. Palmblad M. (2009). Alignment of capillary electrophoresis-mass spectrometry datasets using accurate mass information. *Anal. Bioanal. Chem.* 395 2527–2533. 10.1007/s00216-009-3166-1 19826795

[B22] Nevedomskaya E. Mayboroda O. A. Deelder A. M. (2011). Cross-platform analysis of longitudinal data in metabolomics. *Mol. Biosyst.* 7 3214–3222. 10.1039/c1mb05280b 21947311

[B23] Pacchiarotta T. Hensbergen P. J. Wuhrer M. van Nieuwkoop C. Nevedomskaya E. Derks R. J. E. (2012). Fibrinogen alpha chain O-glycopeptides as possible markers of urinary tract infection. *J. Proteomics* 75 1067–1073. 10.1016/j.jprot.2011.10.021 22075168

[B24] Peng H.-C. Wang Y.-H. Wen C.-C. Wang W.-H. Cheng C.-C. Chen Y.-H. (2010). Nephrotoxicity assessments of acetaminophen during zebrafish embryogenesis. *Comp. Biochem. Physiol. C Toxicol. Pharmacol.* 151 480–486. 10.1016/j.cbpc.2010.02.004 20170747

[B25] Peterson R. T. Macrae C. A. (2012). Systematic approaches to toxicology in the zebrafish. *Annu. Rev. Pharmacol. Toxicol.* 52 433–453. 10.1146/annurev-pharmtox-010611134751 22017682

[B26] Pirmohamed M. James S. Meakin S. Green C. Scott A. K. Walley T. J. (2004). Adverse drug reactions as cause of admission to hospital: prospective analysis of 18 820 patients. *BMJ* 329 15–19. 10.1136/bmj.329.7456.15 15231615 PMC443443

[B27] Portilla D. Li S. Nagothu K. K. Megyesi J. Kaissling B. Schnackenberg L. (2006). Metabolomic study of cisplatin-induced nephrotoxicity. *Kidney Int.* 69 2194–2204. 10.1038/sj.ki.5000433 16672910

[B28] Rider S. A. Tucker C. S. Del-Pozo J. Rose K. N. MacRae C. A. Bailey M. A. (2012). Techniques for the in vivo assessment of cardio-renal function in zebrafish (Danio rerio) larvae. *J. Physiol.* 590 1803–1809. 10.1113/jphysiol.2011.224352 22331420 PMC3573304

[B29] Santangelo F. Drüeke T. Descamps-Latscha B. (2004). Restoring glutathione as a therapeutic strategy in chronic kidney disease. *Nephrol. Dial. Transplant.* 19 1951–1955. 10.1093/ndt/gfh266 15252168

[B30] Shang Y. Siow Y. L. Isaak C. K. Karmin O. (2016). Downregulation of glutathione biosynthesis contributes to oxidative stress and liver dysfunction in acute kidney injury. *Oxid. Med. Cell. Longev.* 2016 1–13. 10.1155/2016/9707292 27872680 PMC5107229

[B31] Shaw J. P. Sueoko C. M. Oliyai R. Lee W. A. Arimilli M. N. Kim C. U. (1997). Metabolism and pharmacokinetics of novel oral prodrugs of 9-[(R)-2-(phosphonomethoxy)propyl]adenine (PMPA) in dogs. *Pharm. Res.* 14 1824–1829. 9453075 10.1023/a:1012108719462

[B32] Slocum J. L. Heung M. Pennathur S. (2012). Marking renal injury: can we move beyond serum creatinine? *Transl. Res.* 159 277–289. 10.1016/j.trsl.2012.01.014 22424431 PMC3308350

[B33] Smith C. A. Want E. J. O’Maille G. Abagyan R. Siuzdak G. (2006). XCMS: processing mass spectrometry data for metabolite profiling using nonlinear peak alignment, matching, and identification. *Anal. Chem.* 78 779–787. 10.1021/ac051437y 16448051

[B34] Sumner L. W. Amberg A. Barrett D. Beale M. H. Beger R. Daykin C. A. (2007). Proposed minimum reporting standards for chemical analysis. *Metabolomics* 3 211–221. 10.1007/s11306-007-0082-2 24039616 PMC3772505

[B35] van Ravenzwaay B. Herold M. Kamp H. Kapp M. D. Fabian E. Looser R. (2012). Metabolomics: a tool for early detection of toxicological effects and an opportunity for biology based grouping of chemicals-from QSAR to QBAR. *Mutat. Res.* 746 144–150. 10.1016/j.mrgentox.2012.01.006 22305969

[B36] Wang W. Ballatori N. (1998). Endogenous glutathione conjugates: occurrence and biological functions. *Pharmacol. Rev.* 50 335–356. 9755286

[B37] Waring M. J. Arrowsmith J. Leach A. R. Leeson P. D. Mandrell S. Owen R. M. (2015). An analysis of the attrition of drug candidates from four major pharmaceutical companies. *Nat. Rev. Drug Discov.* 14 475–486. 10.1038/nrd4609 26091267

[B38] Westhoff J. H. Giselbrecht S. Schmidts M. Schindler S. Beales P. L. Tönshoff B. (2013). Development of an automated imaging pipeline for the analysis of the zebrafish larval kidney. *PLoS One* 8:e82137. 10.1371/journal.pone.0082137 24324758 PMC3852951

[B39] Wyss M. Kaddurah-Daouk R. (2000). Creatine and creatinine metabolism. *Physiol. Rev.* 80 1107–1213. 10.1152/physrev.2000.80.3.1107 10893433

[B40] Xu E. Y. Perlina A. Vu H. Troth S. P. Brennan R. J. Aslamkhan A. G. (2008). Integrated pathway analysis of rat urine metabolic profiles and kidney transcriptomic profiles to elucidate the systems toxicology of model nephrotoxicants. *Chem. Res. Toxicol.* 21 1548–1561. 10.1021/tx800061w 18656965

[B41] Zgoda-Pols J. R. Chowdhury S. Wirth M. Milburn M. V. Alexander D. C. Alton K. B. (2011). Metabolomics analysis reveals elevation of 3-indoxyl sulfate in plasma and brain during chemically-induced acute kidney injury in mice: investigation of nicotinic acid receptor agonists. *Toxicol. Appl. Pharmacol.* 255 48–56. 10.1016/j.taap.2011.05.015 21640743

[B42] Zhang P. Chen J. Wang Y. Huang Y. Tian Y. Zhang Z. (2016). Discovery of potential biomarkers with dose- and time-dependence in cisplatin-induced nephrotoxicity using metabolomics integrated with a principal component-based area calculation strategy. *Chem. Res. Toxicol.* 29 776–783. 10.1021/acs.chemrestox.5b00519 27030963

