# Supplementary material for: Zebrafish Larvae Are a Suitable Model to Investigate the Metabolic Phenotype of Drug-Induced Renal Tubular Injury
Source: Front Pharmacol. 2018 Oct 16;9:1193. doi: 10.3389/fphar.2018.01193 (PMC6232664; doi:10.3389/fphar.2018.01193)
Supplement: Supplementary file 1 [file Data_Sheet_1.PDF]

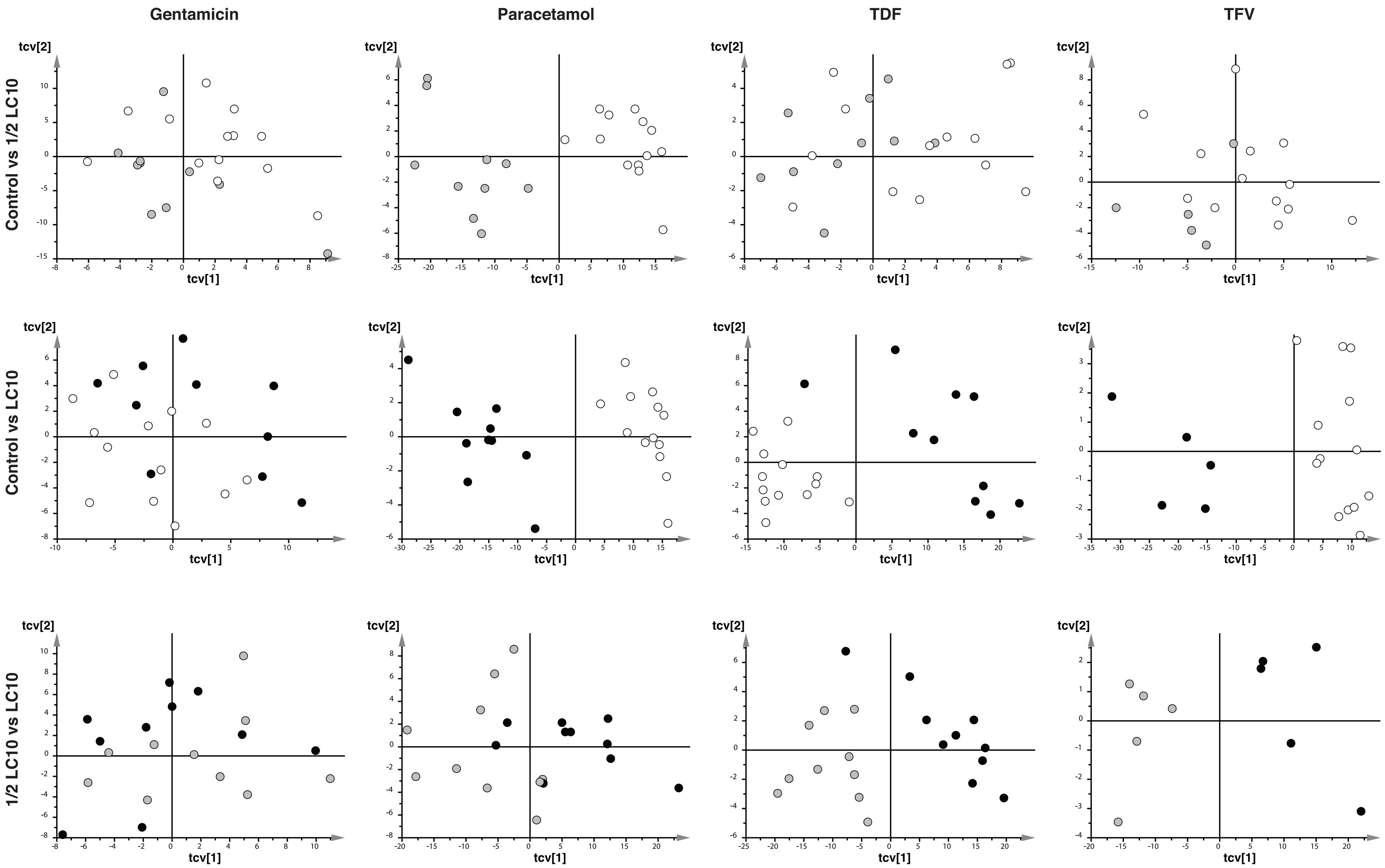

Supplementary Figure 1. Cross Validated score plots of the PLS-DA models between controls and ½ LC10, controls and LC10 and ½ LC10 and LC10 for gentamicin, paracetamol, TDF and TFV. White dots correspond to controls, grey dots to zebrafish exposed to ½ LC10 and black dots to zebrafish exposed to LC10.
