# Supplementary material for: Zebrafish Larvae Are a Suitable Model to Investigate the Metabolic Phenotype of Drug-Induced Renal Tubular Injury
Source: Front Pharmacol. 2018 Oct 16;9:1193. doi: 10.3389/fphar.2018.01193 (PMC6232664; doi:10.3389/fphar.2018.01193)
Supplement: Supplementary file 2 [file Data_Sheet_2.PDF]

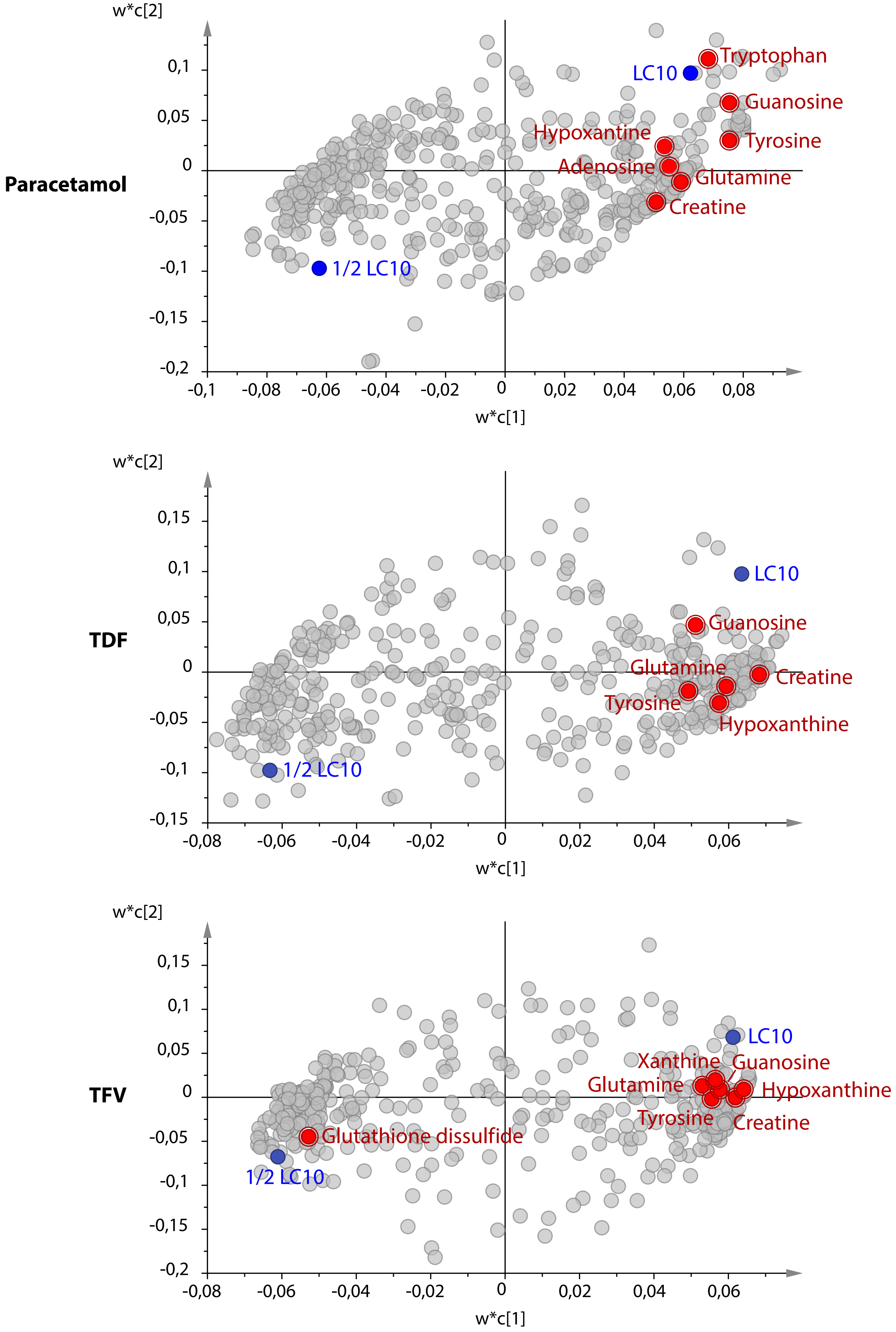

Supplementary Figure 2. Loadings scatter plots of the PLS-DA models between ½ LC10 and LC10 showing the relationship between the Y-variable and the X-variables. Y-variable (½ LC10 versus LC10) is colored in blue. X-variables (metabolites) are colored in grey except the ones that were identified, which appear in red and with their respective identification name.

TDF: tenofovir disoproxil fumarate; TFV: tenofovir
