# Supplementary material for: Zebrafish Larvae Are a Suitable Model to Investigate the Metabolic Phenotype of Drug-Induced Renal Tubular Injury
Source: Front Pharmacol. 2018 Oct 16;9:1193. doi: 10.3389/fphar.2018.01193 (PMC6232664; doi:10.3389/fphar.2018.01193)
Supplement: Supplementary file 3 [file Table_1.docx]

**Supplementary table 1. List of excluded exogenous compounds.**

Assignments of drug and drug metabolite were described in our previous article (Gorgulho et al. 2017).

| **Retention time (secs)** | ***m/z*** | **Ion** |
| --- | --- | --- |
| 189.93 | 110.0601 | [Paracetamol+H-H_2_O-C_2_]^+^ |
| 190.09 | 152.0707 | [Paracetamol+H]^+^ |
| 189.87 | 153.0740 | [Paracetamol+H+1*]^+^ |
| 189.76 | 171.0441 | Unknown |
| 190.09 | 174.0526 | [Paracetamol+Na]^+^ |
| 190.34 | 175.0557 | [Paracetamol+Na+1*]^+^ |
| 190.59 | 180.0494 | Unknown |
| 105.39 | 194.0619 | Unknown |
| 105.48 | 210.0619 | Unknown |
| 190.96 | 236.0229 | Unknown |
| 105.39 | 236.0779 | Unknown |
| 41.08 | 239.1062 | [HEPES+H]^+^ |
| 189.90 | 246.5761 | Unknown |
| 190.08 | 248.0934 | Unknown |
| 190.96 | 254.0334 | Unknown |
| 40.05 | 277.0619 | [HEPES+K]^+^ |
| 75.37 | 288.0859 | [TFV+H]^+^ |
| 75.31 | 289.0885 | [TFV+H+1*]^+^ |
| 75.48 | 310.0675 | [TFV+Na]^+^ |
| 153.11 | 328.1024 | [Paracetamol glucuronide+H]^+^ |
| 153.29 | 350.0843 | [Paracetamol glucuronide+Na]^+^ |
| 69.12 | 365.1280 | Unknown |

* Second isotope.

**Supplementary table 2. Parameters of PLS-DA models for the 2 first components.**

| **PLS-DA model** | **Drug** | **R2(x)cum** | **R2(y)cum** | **Q2(y)cum** | **p-value** |
| --- | --- | --- | --- | --- | --- |
| Controls vs ½ LC10 | Gentamicin | 0.299 | 0.897 | 0.514 | >0.1 |
|  | Paracetamol | 0.553 | 0.959 | 0.903 | 1.27x10^-6^ |
|  | TDF* | 0.148 | 0.584 | 0.232 | 0.07 |
|  | TFV | 0.313 | 0.92 | 0.451 | >0.1 |
| Controls vs LC10 | Gentamicin | 0.357 | 0.782 | 0.403 | 0.09 |
|  | Paracetamol | 0.635 | 0.982 | 0.932 | 3.89x10^-10^ |
|  | TDF | 0.529 | 0.947 | 0.852 | 2.38x10^-5^ |
|  | TFV | 0.59 | 0.97 | 0.886 | 2.10x10^-5^ |
| ½ LC10 vs LC10 | Gentamicin | 0.303 | 0.848 | -0.0317 | >0.1 |
|  | Paracetamol* | 0.378 | 0.566 | 0.41 | 0.011 |
|  | TDF | 0.51 | 0.95 | 0.824 | 2.99x10^-4^ |
|  | TFV | 0.62 | 0.978 | 0.848 | 0.023 |

* Only 1 component

**Supplementary table 3. Identified metabolites after MS/MS experiments.**

| **Class** | **Metabolite** | **Retention time**  **(secs)** | ***m/z***  **[metabolite+H] ^+^** | **MS/MS fragments** | **Drug** |
| --- | --- | --- | --- | --- | --- |
| Purine metabolites | Adenosine | 77.39 | 268.1041 | 136.0617 | Paracetamol |
|  | Guanosine | 96.31 | 284.0991 | 152.0569  135.0297 | Common |
|  | Hypoxanthine | 62.96 | 137.0459 | 137.0459  119.0348 | Common |
|  | Xanthine | 72.20 | 153.0409 | 136.0132  110.0343 | Tenofovir |
| aa and peptides | Creatine | 42.12 | 132.0768 | 90.0550  44.0498 | Common |
|  | Glutamine | 42.12 | 147.0765 | 130.0501  84.044 | Common |
|  | Glutathione dissulfide | 65.98 | 613.1589 | 484.1159  335.0742  231.0444  185.0379  130.0500 | Tenofovir |
|  | Tryptophan | 183.68 | 205.0973 | 188.0709  146.0598  115.0542 | Paracetamol |
|  | Tyrosine | 64.94 | 182.0813 | 165.0547  147.0442  136.0759  123.0439  119.0492  65.0387  51.0229 | Common |
